# Health Literacy in the Everyday Lives of Older Adults in Greece, Hungary, and the Netherlands

**DOI:** 10.3390/ijerph17072411

**Published:** 2020-04-02

**Authors:** Liesbeth de Wit, Pania Karnaki, Archontoula Dalma, Peter Csizmadia, Charlotte Salter, Andrea de Winter, Louise Meijering

**Affiliations:** 1 Population Research Centre/Urban and Regional Studies Institute, University of Groningen, 9700 AV Groningen, The Netherlands; liesbeth.s.dewit@gmail.com; 2 Institute of Preventive Medicine, Environmental & Occupational Health, Prolepsis, Marousi, 15125 Athens, Greece; p.karnaki@prolepsis.gr (P.K.); n.dalma@prolepsis.gr (A.D.); 3 National Institute for Health Development (OEFI), 1096 Budapest, Hungary; csizmadia.peter@oefi.antsz.hu; 4 Norwich Medical School, University of East Anglia, Norwich NR4 6NY, UK; c.salter@uea.ac.uk; 5 Department of Health Sciences, University of Groningen, University Medical Center Groningen, 9700 AD Groningen, The Netherlands; a.f.de.winter@umcg.nl

**Keywords:** critical health literacy, lay perspectives, older adults, health care professionals, health care system, qualitative methods

## Abstract

Health literacy (HL) encompasses someone’s knowledge and abilities to access and use health information in order to make appropriate health decisions in life. HL is particularly valuable in later life when health challenges grow. An individual’s HL is typically considered a fixed and skills-based characteristic, without taking into account how these are situated in the context of everyday life. Also, lay perspectives on health literacy are relatively scarce. Therefore, the aim of this article is to explore the context-specific perspectives of older adults and health professionals on HL in later life in Greece, Hungary, and the Netherlands. We adopted a qualitative methodology and conducted 12 focus groups: seven with 50 older adults and five with 30 health professionals to gain insight into individual perspectives on HL as situated in the health care and everyday life contexts. An informed grounded theory approach was used in analyzing the data. The results are structured in three themes: (1) interactions with health professionals, (2) perceived quality of the health care system, and (3) managing health in the context of everyday life. An overarching finding is that, for older adults, HL reflects the demands placed on them when managing their health. In the experience of older adults, these demands are placed upon them by healthcare professionals, the healthcare system, as well as their everyday lives. Our findings underscore the importance of Critical Health Literacy (CHL) as that concept foregrounds that HL is context specific. Also, CHL has been argued to be a community characteristic, which is why we call for community-based approaches to improve HL.

## 1. Introduction

Health literacy is an important topic in health research as it contributes to health and well-being of individuals and community [1]. Following advances in health literacy (HL) made in the USA, Canada, and Australia, in the past two decades, HL has become a public health issue of interest to researchers, practitioners, and policy-makers in Europe [2,3]. Health literacy is a broad concept and has been defined as covering “people’s motivations, knowledge, and competences to access, understand, appraise, and apply health information in order to make judgments and take decisions in everyday life concerning healthcare, disease prevention, and health promotion to maintain or improve quality of life during the life course” [4] (p. 3). As such, HL incorporates different motivations, different types of knowledge and skills, and different types of health information at different timings and contexts in people’s lives and about different health and welfare topics. 

Research has shown that, in Europe, older adults in particular are at risk of having relatively low levels of HL due in part to their increased risk of having health problems and in part to the inherent complexities of managing health [5,6]. There is evidence that lower HL abilities are associated with poorer health outcomes [7], dementia [8], and higher mortality rates in later life [9]. It has also been shown that older adults with higher HL abilities are better able to manage their illnesses as well as their overall health and well-being [10]. 

The majority of HL initiatives aim to improve health outcomes by addressing the knowledge and skills of patients or health professionals in clinical settings. The initiatives focus, for instance, on improving comprehension of health recommendations, self-management of disease and health, or communication between patients and health professionals [11,12,13]. In addition, several HL initiatives aim to improve the health of socially excluded populations through education initiatives addressing contextual factors that influence health [14]. Overall, most HL interventions try to improve patients’ skills to comprehend health messages and recommendations as well as to better interact with health professionals. As a result of this focus on improving skills, the knowledge of people with regard to their health, as embedded in the contexts of their everyday lives, is often overlooked. This is an important gap, as both the perspectives of such “lay people” and the role of context are an integral part of HL. There is some research on both components but not a lot. 

When looking into research into the perspectives of people on HL, the work of Thomas Abel is important to discuss. According to Abel [15,16,17], perspectives of “ordinary” people, or so-called lay perspectives on HL, find their origin in the knowledge people have accrued from their life experiences of health and illness in the context of their everyday lives. Using this definition, lay perspectives may include experiences of people who are healthy as well as those who are (chronically) ill. A couple of studies have explored people’s perspectives on HL, often focusing on those who are (at risk of becoming) ill. This includes work by Edwards et al. [18], who conducted a longitudinal qualitative study on how patients with a long-term health condition became more health-literate over time in the UK, and by Van Onna et al. [19], who studied HL in patients with gout in the Netherlands. Furthermore, there are studies on HL of diabetic Chinese immigrants in the US [20] and on HL of persons at risk of becoming chronically ill in Germany [21].

Furthermore, there are a few studies that compare perspectives of different groups of people on HL, such as Salter et al. [22], who studied people with chronic musculoskeletal conditions, health professionals, and caretakers in the UK, and Jordan et al. [23], who studied people with a chronic illness, people who had been treated at an emergency ward, and the general public in Australia. Finally, there are studies on people’s perspectives on HL that include a subset of the population, such as adult learners, older adults, or migrants [24,25,26]. All these studies on people’s perspectives on HL explored a range of settings, often healthcare settings or a specific setting in people’s lives (such as a learning environment) but not in their everyday lives. The overarching findings of these studies indicate that people perceive the interactions between individuals and their social and health care environments as one of the most important aspects of HL. However, while the context of an individual’s life is essential to a lay perspective, none of the abovementioned studies explored people’s context in a broader sense, that is, the context of their everyday lives and the context of the combined health care settings in which they participate. Furthermore, there are, to our knowledge, no studies that look into people’s perspectives on HL in different countries.

There have been a number of recent studies on HL in context [10]. These studies looked into, for instance, maternal HL [27], HL of young people in relation to type 2 diabetes [28] and alcohol use [29], barriers to breast cancer screening [30], and HL in relation to characteristics of welfare states in Europe [31]. Most of these recent studies on the role of context in HL use a quantitative approach and therefore do not consider the perspectives of people in much depth. Exceptions are the studies by Thomas et al. [27] and Schillinger et al. [28], who carried out a mixed-methods study on the social conditions and dynamics affecting maternal health literacy and a qualitative study evaluating a public health campaign to raise awareness of type 2 diabetes among young people, respectively. Furthermore, the importance of cultural context has been foregrounded in studies on migrant HL [20,26]. Overall, these studies on context bring to light that individual, social, economic, psychological, and environmental factors can all affect how individuals manage their health and, in that sense, their HL. These two areas of research, on “ordinary” people’s perspectives on HL and on the role of context in HL, do provide emerging perspectives beyond that of health professionals and the clinical setting, each in their own way. However, people’s and health professionals’ perspectives on HL as situated in the context of everyday life have not been studied. 

As a result, it is still largely unclear to what extent HL initiatives’ focus points correspond to those of “ordinary” people. Perspectives of people and health professionals on HL will increase our understanding of the motivations, knowledge, skills, and information that people use to manage their health in their everyday lives. Such insights will help to better target HL initiatives towards the needs of the people concerned [32]. This study contributes to the identified knowledge gaps and aims to explore the perspectives of older adults and health professionals on HL in later life in Greece, Hungary, and the Netherlands. The perspectives of older adults mainly provide insight into experienced motivations and information, while the perspectives of health professionals provide insight into perceived knowledge, skills, and information. 

## 2. Materials and Methods 

### 2.1. Approach and Study Setting

This study was carried out within the framework of the EU-funded project Intervention Research on Health Literacy among the Aging population (IROHLA). IROHLA aimed to improve the health literacy of adults aged 50 years and older with poor health literacy in Europe [33]. To gain insight into a range of perspectives of older adults and health professionals, we adopted a qualitative approach using focus group discussions (FGDs). FGDs were held as they are well suited to collect a broad range of perspectives of a diverse study population through discussion. Such discussions facilitate extensive and interactive expressions of participants’ views [34]. 

Twelve FGDs were conducted in less-privileged urban neighborhoods with relatively high levels of socioeconomic deprivation in Greece (Athens), Hungary (Budapest), and the Netherlands (Groningen). Since poor HL is associated with low socioeconomic status and the IROHLA project’s focus was on older adults with poor HL, our study participants were recruited from socioeconomically deprived neighborhoods. The three countries were selected for two reasons: (1) the geographical, social, cultural, and health care contexts of these settings differ, and (2) partners in the IROHLA project in these countries possessed expertise on conducting qualitative research. As a result, we were able to explore a wide range of perspectives on HL in later life. Seven FGDs were conducted with 50 community-dwelling adults aged 50 or older. This was the same age-group as in the overall IROHLA project. In IROHLA, we decided to start inclusion at this relatively low age since people with low HL often have (multiple) diseases and since mortality is relatively high. In addition, to gain further insight into older adults’ HL, five FGDs were conducted with 30 health professionals who were working in these older adult communities (see Table 1). These health professionals were employed as social workers, nurses, general practitioners, and medical specialists. 

The research team consisted of researchers who had previous experience with conducting qualitative research and were from the University of Groningen in the Netherlands, and two nonprofit organizations specialized in public health and health promotion in Greece and Hungary. Regular skype meetings and debriefs were organized throughout the study to ensure that the team had a common understanding of the research process and practices. For the recruitment of participants, we used the partner organization’s networks as well as community gatekeepers. Gatekeepers are individuals or organizations who have a prominent role in the community. [34]. In line with this strategy, a suitable way for recruiting participants was chosen in each country, based on its cultural and institutional context (see Table 2). Inclusion criteria were (1) being aged 50 years and over and (2) living in deprived urban areas. We aimed at a high diversity in gender, health conditions, and living situation. For health professionals, the inclusion criterion was that they were working with this particular population group. 

### 2.2. Data Collection

In the FGDs with older adults, we tried to capture all aspects of HL as defined by Sørensen et al. [4]. In so doing, we developed two FGD guides, one for the older adults and one for the health professionals (see Supplementary Materials). In the FGDs with the older adults, we discussed three main topics: (1) perception of health and well-being, (2) coping with health and experiencing health services, and (3) health information. In the FGDs with health professionals, we focused on the second and third topics. In all FGDs an open approach with broad questions and probes was used. These gave us insight into participants’ motivations, knowledge, competences, and use regarding the topics that were discussed, thus generating rich perspectives on HL. The discussion guide was developed in English, translated into the local languages, and pilot-tested in each country. For the third topic, two posters prompted further discussion. Each of the posters represented a health risk and a health promotion topic. The posters had been developed as health information in other studies and had been used in Hungary and the Netherlands. We translated them into English and the local languages and pilot tested them in each country. The posters are available on request. In all FGDs, a moderator led the discussion and encouraged the participants to talk and interact with each other. A second researcher took notes and supported the facilitation. At the start of the FGDs, the procedures and the participants’ rights were explained to the participants; time for questions was provided. The duration of one FGD was about 90 min. To validate the researchers’ interpretations of the FGD data, follow-up FGDs were organized with the participants who were willing and able to participate once more, which was the majority. In these follow-up FGDs, the findings of the FGDs conducted in the specific country were presented and discussed with the participants. The majority of the original participants joined in person, but three health professionals in the Netherlands gave input through an email questionnaire because they were not able to come to the meeting.

Ethical approval to conduct the study (project identification number 305831) was obtained in each country in accordance with local procedures and guidelines. In the Netherlands, the Research Ethics Committee, Faculty of Spatial Sciences, University of Groningen approved the study on 11/02/13. In Hungary, the Ethics Committee of the National Institute of Health Promotion approved the study on 21/05/13. In Greece, approval by an IRB or local ethics committee was not required as the study did not involve any human or animal testing. A common consent form was developed by the team prior to the study as part of the research protocol. Then, this was translated into the local languages. Participants gave written informed consent prior to the FGDs.

### 2.3. Data Analysis

The FGDs were recorded and transcribed verbatim by the researchers. All identifiers were removed from the transcripts to anonymize the data. We used a common data-analysis protocol in all three countries. The transcript of the first FGD conducted in each country was translated into English, and each country partner coded all three transcripts using a preliminary codebook that was developed based on the discussion guide and the theoretical framework. The researchers then compared the codes, discussed the coding methods, and agreed upon a shared codebook. Further coding was done separately in each country and in the local language using the final codebook, which contained the codes and a brief description of the meaning of each code. If, however, the researchers felt that a new code had to be used, it was added to the codebook iteratively. We used an informed grounded theory approach to analyze the data. This approach is characterized by combining the principles of grounded theory and analysis informed by previous literature and theory [35]. Here, the broad health literacy concept served as a starting point in the analysis that included the topics in the discussion guide: (1) health and well-being in the context of life, (2) managing health and disease, and (3) health services and health information. The HL concept was then extended through the themes that emerged from the data according to the principles of grounded theory [34]. The themes identified in each country were discussed by the research team, and additions were made based on the follow-up FGDs. The first author analyzed the final results from the three countries, sought for common subthemes, and discussed these with the researchers from the other countries until agreement was reached.

## 3. Results

### 3.1. Participant Characteristics

The characteristics of the older adults who participated in the FGDs are presented in Table 3. The participants in Greece were generally older than those in Hungary and the Netherlands. The education levels of the older adults were, on average, lower in Greece than in Hungary and the Netherlands. In Greece and in Hungary, a majority of the older adults were retired, whereas in the Netherlands, only half of the older adults were retired. On average, the Dutch participants had less health problems and better self-reported health than the Hungarian and Greek participants.

With regard to the participating health professionals, it is noteworthy that more health professionals participated in Greece and in Hungary than in the Netherlands (Table 4). Besides, in Greece and in Hungary, more health professionals were working in a clinical setting than in the Netherlands, where more community health workers participated. Professionals working in clinical settings were approached in the Netherlands, but they indicated that they did not have time to participate in the study.

### 3.2. Interactions with Healthcare Professionals

In all three countries, the context in which older adults interact with health professionals emerged as an important setting that affected how our participants managed their health. For instance, the doctor’s consultation room was mentioned as an important place where the participants got medical advice. With use of the word “doctor”, participants typically referred to their family physician or general practitioner. Overall, our participants said they strongly value the medical advice from a knowledgeable health professional, such as the general practitioner. Preferences with regard to how consultations with health professionals are structured and organized proved to differ between the countries, however. In the Netherlands, the majority of older adults articulated having a rather large share in the conversation with health professionals. Some participants believed it is their responsibility to be assertive in interactions with health professionals because doing so ensures that they are involved in decision-making:

*I very much depend on care, but I should not act like a dependent person. Nowadays, one should be assertive*.

(Dutch older adult)

Besides the importance of being assertive, Dutch participants also emphasized that empathy and trust are crucial values for successful communication between patient and health professional. However, they were adamant that this trust relationship did not always exist, in which case, they felt the quality of the interaction with a health professional was low. 

In Hungary, some older adults said they ask questions to their health professionals while others felt afraid to do so. In the latter case, participants reported that health professionals often did not respond to their silences. As a result, they felt health professionals do not take the initiative in explaining what is at hand. Rather, they only respond to questions asked by patients. A health professional confirmed he did not encourage dialogue with his patients:

*I can only respond to the questions that they ask me*.

(Hungarian health professional)

As a result of this, our Hungarian participants felt they could not share their experiences and health problems fully with their health professionals. In line with this, a recurring issue in the discussions was how participants experienced difficulties with getting a health professional’s full attention. Many participants said, for instance, they try to get the doctor’s time and attention by bringing gifts in the form of a present or money. Overall, our Hungarian participants felt they were not taken seriously, not sufficiently informed, and reported they distrust health professionals in general. 

In Greece, in contrast, the fact that patients shared little in conversations with health professionals seemed to be the norm. While the Greek participants generally agreed that patient input is needed to make a correct diagnosis, a majority of the Greek older adults said they never question the health advice of health professionals, doctors in particular. They said they have great respect for their doctor and expect him or her to ask them the right questions:

*It’s the role of the doctor to ask the patient everything. Whether the patient provides the answer or not depends upon each [patient]*.

(Greek older adult)

This quote illustrates that our Greek participants see it to be the role of the health professional to get all the information they need from a patient. In line with this, our Greek participants discussed that it was difficult for them to open up to health professionals. One participant explained how she needed the undivided attention of a medical specialist to be able to open up:

*There was a problem with the doctor’s PC and she was not able to write the prescription, so she was occupied with solving the problem. She said to me, “keep talking, I’m hearing you.”, but I couldn’t say anything to her; since she was busy, I shut down. I told her only one thing, and not all the information that I had written down*.

(Greek adult)

In all three countries, the majority of older adults experienced insufficient time to discuss their health issues with health professionals due to the short consultation times, which resulted in communication problems between, for instance, patient and doctor. In all countries, professionals experienced that they were not provided with sufficient consultation time by their health care system to enable successful communication. In the different countries, communication problems between patient and health professional were associated with different cultural values and normative behaviors. Several Greek and Hungarian healthcare professionals acknowledged that short consultation times do not allow doctors to carefully listen to their patients and that their ability to offer health advice is therefore curtailed:

*There used to be information leaflets in the waiting room, and older adults came with the leaflet and asked me about it during the appointment. It was not possible for me to answer all these questions and make the diagnosis in the space of only 10 min, so I hid the leaflets*.

(Greek health professiona)

This quote illustrates how health professionals in both Greece and Hungary have limited time to spend with each patient, which does not allow them to discuss topics beyond what the patient has come to see them for.

### 3.3. Perceived Quality of the Health Care System

The health care system is another setting that our participants reported to be important. It is important to acknowledge the differences between the health care systems of the three countries. In Greece, the health care system consists of public and private health services. Greek pensioners are fully covered by the public health care system but pay 10 percent of the costs of prescriptions, doctor’s visits, and treatments. Hungary has a tax-funded health care system. Health care for pensioners is free of charge except for certain services, such as rehabilitation and some medications. In the Netherlands, having a basic health insurance is compulsory. The basic insurance covers the most common treatments and medications and costs around €100 a month. In addition, all adults have to pay the first €375 they spend on healthcare out of their own pocket. Beyond that amount, healthcare is free. Additional service packages beyond basic insurance can be purchased at monthly fees ranging around up to €40.

In all three countries, participants shared their views on the quality of the health care system. Overall, as opposed to the Dutch participants, the majority of Greek and Hungarian participants perceived the quality of the health care system and health services offered as low. This resulted in dissatisfaction with the system and people hesitating to use the health services available to them. In relation to the quality of health services, older adults in all three countries said that they find adequate health care is too expensive. The high cost of health care limited many of our participants in choosing the health services and treatments that would be most appropriate for their needs. Because of the differences in health system, the participants’ experiences in this regard differed slightly between the three countries.

In Greece, many of the older adults said they would prefer to use private health services, which they believe are of higher quality than public services. However, most of the participants could not afford private health care:

*[You] are afraid, and you think that, if you don’t have money, you have to deal with doctors in the [public health care system] and that is terrible*.

(Greek older adult)

This comment illustrates that many older adults have no choice but to use public health services that they perceive to be of inferior quality.

In Hungary, several of the older adults articulated how a lack of affordability influenced their health care choices. Some older adults described they sometimes do not take their prescribed medications because they cannot afford them. Furthermore, they explained how they avoid making an appointment with a doctor because they cannot afford the customary gift. Several health professionals recognized this behavior and reported that such avoidance of care could worsen the long-term health of older adults:

*They won’t call the doctor because they feel awkward about not being able to give any money*.

(Hungarian health professional)

In the Netherlands, several of the adults in the study said that, while the health care they receive is adequate, it is very expensive:

*Health care is very good here, compared to other countries, but it is extremely expensive*.

(Dutch older adult)

Some of the participants chose to pay for the most expensive supplementary scheme on top of their basic health insurance to be covered for potentially expensive treatments. In such cases, a large portion of the adult’s relatively low income was spent on health care. Several of the health professionals said they recognize that being unable to afford a comprehensive health insurance is a problem:

*Particularly for older adults with low incomes, this is a difficult problem*.

(Dutch health professional)

In all three countries, access to health services was a topic that dominated the discussions in the different countries in different ways. In Greece and Hungary, many participants experienced unclear directions for obtaining health services in hospitals and experienced long waiting lists for doctor’s appointments. How these issues could have serious health consequences was explained by one of the Hungarian participants:

*I have to wait for months for a CT scan or MRI; I might not even live that long*.

(Hungarian older adult)

In addition to long waiting times, Hungarian adults also mentioned they experience problems with the coordination between specialists. They told us that multiple treatments and medication prescriptions are often not sufficiently aligned, causing extensive time spent waiting and travelling. This lack of coordination was a burden, especially for the less mobile older adults. 

In the Netherlands, some of the older adults mentioned that they have to choose a health and social care provider from a large number of services. Being responsible for these decisions made it difficult for them to identify and obtain the appropriate care. Among them, too, this led to dissatisfaction with the healthcare system:

*It would be better if there was one place where all [organizations] were based*.

(Dutch older adult)

The quote raises the issue of fragmentation of health care, a problem which several of the Dutch health professionals acknowledged:

*There is a jungle of organizations that all strive for profit and strongly compete with each other. Making choices does not become easier because of this.*.

(Dutch health professional)

In Hungary, many participants also said they find the health information system too complex. The main problem identified was an excessive amount of health information that is insufficiently aligned across multiple channels. As a consequence, ambiguous health messages and a lack of public trust in the overall health care system occurred. One of the older adults expressed a desire for a solution to this problem:

*Someone should coordinate this [ambiguity of information]; the best would be the GP*.

(Hungarian older adult)

To sum up, our participants reported the health care systems in which they have to find their way as complex, expensive, and difficult to access. As a result, they often do not seek the care they know they would need. Also, although experiencing problems with navigating the health care systems first-hand, our participants did not feel empowered to change these systems but rather felt they had to endure them.

### 3.4. Managing Health in the Context of Everyday Life

The third context in which our participants managed their health was their everyday life. Our participants seemed to see this context as most important, and the majority offered explanations on how they manage their health as part of their everyday lives. These were mainly articulated by a strong willingness to care for oneself as much as possible and participation in activities that support health and well-being.

In all three countries, the ability to care for oneself was a major discussion topic, although it was discussed in different ways. In Greece and in Hungary, where the majority of participants faced one or more health problems, participants particularly talked about their fear to lose the ability to care for themselves when physical or mental health would decline in the future. As in Greek and Hungarian culture a large pressure lies on the family to offer full support when a family member becomes dependent on care, the majority of Greek and Hungarian participants, irrespective of the severity of their health problems, did not want to become a burden for their family:

*Because of asthma, I find carrying heavy loads difficult but I don’t want to bother my children, so I manage on my own*.

(Greek older adult)

Although the Greek participants in particular strongly articulated that they wanted to avoid burdening their family, the oldest generation of adults expressed they expect their children to care for them in the future:

*I don’t think about a residential care home; I don’t want [to live in] a residential care home. I looked after my mother until she passed away*.

(Greek older adult)

Unlike Greece and Hungary, in the Netherlands, where the majority of the adults faced less health problems, being able to care for oneself was talked about by the majority of participants as being something self-evident and necessary for health and well-being. Many Dutch participants also articulated the need to be able to make their own choices regarding their health and well-being:

*I can’t stand it if people are deciding for me*.

(Dutch older adult)

In all three countries, participants expressed that being financially independent was an important part of their ability to care for themselves. In Greece and Hungary, many of the older adults mentioned being anxious about financially burdening their family but that they may not be able to avoid asking for financial support because of their low pension levels.

*It’s important for people to maintain their health status somewhat. They should be able to do the daily routine things, even to earn some money,… to be in a state where they do not depend on anyone’s help*.

(Hungarian older adult)

In all three countries, another dominant discussion topic contained participation in activities that support health and well-being. As part of this subtheme, our participants also stressed the importance of having a strong social network, including family as well as friends and acquaintances. An important activity that they engaged in was family life. Participants strongly articulated that being actively involved in family life, for instance by caring for grandchildren, significantly contributed to their health and well-being. Family deemed to play an essential role in the health and well-being of the Greek older adults in particular:

*There is no life without family*.

(Greek older adult)

While the Greek participants placed strong emphasis on the importance of family life for their health and well-being, they did not talk about the importance of organized activities. This was brought forward by participants in the other two countries, however. In Hungary, many participants believed that they needed to participate in organized activities to maintain their health and well-being. Some Hungarian adults said to do this through exercising, attending a choir or needlework group, or mentoring students. In the Netherlands, where most participants faced relatively few health problems, the majority of participants were aware that they could play an active role in maintaining their health and well-being by partaking in organized activities. Many of them were engaged in various organized activities to support their physical, mental, and social health and well-being, such as cycling, gardening, fishing, cooking, and having communal dinners. Some of the Dutch adults who were not yet retired emphasized that the paid work they did contributed to their social health and well-being as it provided them with social contacts and opportunities to feel useful and to help others.

## 4. Discussion

This article aimed to explore the context-specific perspectives of older adults and health professionals on HL in later life in Greece, Hungary, and the Netherlands. These perspectives revealed three important contexts in which older adults manage their health: (1) interactions with health professionals, (2) the health care system, and (3) their everyday lives. An overarching finding is that HL reflects the demands placed on older adults when they are managing their health. In describing the demands placed on them by healthcare professionals, the healthcare system, and everyday life, the older adults in our study reported a strong need to be in control of their own health and well-being. They also explained how being in control is difficult in each of the three contexts. These results are summarized in Table 5.

With regard to the first theme of our results, we found that, when interacting with health professionals, some older adults, especially in the Netherlands and Hungary, did feel empowered to engage in conversations. However, other participants experienced that health professionals were often not listening or not prioritizing the concerns voiced by older adults. Typically, this appeared to be caused by a lack of time that health professionals have to interact with patients. Our evidence suggests that health care professionals only have the time to concisely discuss a particular health problem with their patients. As a result, a more holistic picture of a patient’s health and well-being may be overlooked.

When looking at the health care system in more general terms, which is the second theme in our results, there were barriers that hindered our participants from being in control of their own health and well-being. In all three countries, financial barriers were discussed. Furthermore, the general complexity of the system and lack of integration and communication between different health care services negatively impacted our participants’ sense of control. In addition, in Hungary and Greece, the poor quality of the public health care system was lamented. 

The third theme, the context of everyday life, was perhaps the context where older adults experienced most control. Although many were afraid of having to become dependent on their children, neighbors, and friends, our participants did take pride in remaining able to look after themselves and to maintain good health and well-being. Our findings thus provide empirical support for Abel’s [16] theoretical argument that people’s knowledge in particular contributes to people’s HL as part of the competence to have control over their health.

The discussion of our findings above demonstrates that the perspectives of older adults and health professionals do provide insight into HL in later life. Our results provide insights around older adults’ motivations to apply health information and how they make decisions around healthcare, disease prevention, and health promotion. These topics are connected to the concept of critical health literacy (CHL). CHL was established as part of general HL by Nutbeam [36] and covers people’s knowledge and abilities to gain and use health information in the context of one’s life in order to exert greater control over one’s health and well-being [37,38,39,40,41]. Our results align with this concept, firstly because they foreground the essential notion of keeping control over one’s health. Secondly, the key role of the context of life that is central to CHL was confirmed by our study. We found several examples that showed how older adults use CHL capacities when interacting with healthcare professionals and in the context of their everyday lives. These examples, which were discussed in the Results section, include engaging family, asking questions, and developing informed decisions with regard to one’s living environment such as residential care. At the same time, we found examples of older adults not being able to use their CHL abilities and when interacting with health professionals, but especially in the context of the health care system. This was related to, for instance, (perceived) financial barriers, lack of time, as well as system-related barriers around arranging care and support. These findings demonstrate that older adults’ contexts determine to a significant extent whether they are enabled to use certain CHL knowledge and abilities.

Our study foregrounded the need to maintain control over one’s own health and well-being. Examples of this need and that of the added value of the perspectives on HL in later life that we studied include that older adults need the full attention of a health professional to open up and that they enjoy being engaged in (organized) activities to maintain health and well-being, such as gardening and exercising. Other research in this field did not report explicitly on a perceived need to maintain control over health and well-being. However, the importance of a needs-based approach in more general terms has been argued for before [22]. Furthermore, several previous studies found aspects that are related to the need for control that we found. This includes the perceived importance of sharing responsibilities with health professionals [22], the need to be assertive in interactions with doctors [23], and the role of motivation in facilitating the development of HL [18,21]. With regard to experienced barriers in using the health care system, other studies found some similar ones, including time constraints in doctor consultations [22,23] and poor communication with health professionals [18,20,26]. 

Building on the finding that the context of everyday life and the healthcare context play an important role in HL, our study showed that HL differs between countries. For example, the older adults in different countries used their health knowledge and abilities in different ways to get their doctor’s attention. In the Netherlands, older adults used assertiveness; in Hungary, they sometimes provided gifts; and in Greece, they behaved passively because they believe it is the responsibility of the doctor to give them the necessary attention. This is in line with Rowlands et al. [24], who found that the cultural context plays a role in how adults evaluate their health knowledge. Overall, our findings around the role of context in HL are in line with the results of previous research, which shows that people’s context determines the meaning and shape of HL [15,38,39].

### 4.1. Limitations 

This article has been one of the first to explore perspectives of older adults and health professionals on HL in later life in three European countries. As they are qualitative in nature, the findings of our study cannot be generalized to a larger population but they are transferable to other settings and target groups as long as the context-specific characteristics of those settings and groups are taken into account. Although there are strengths to our approach, it also has three limitations. First, in the Netherlands, only one group of health professionals participated. Should we have been able to recruit a second group, we would have obtained a richer picture of health professionals’ views on HL in later life. Second, to facilitate discussions with “ordinary” older adults about the complex concept of HL, we operationalized HL by splitting the broad HL definition of Sørensen and colleagues [4] into topics that our participants could manage. These included managing illness and health, health services, and health information in a broad life context. This operationalization may not fully capture the HL concept, for example, older adults’ ability to access and/or understand health information online. However, the approach we used in this study provided us with valuable empirical evidence on people’s perspectives on HL. Third, the characteristics of our participants differed slightly between countries. Compared to the other countries, the participants in Greece were of relatively high age, while the Dutch participants were in relatively good health. These specific characteristics may have contributed in part to differences between countries that we found. Furthermore, the involvement in the study of more health professionals from clinical settings in Greece and Hungary than in the Netherlands may have contributed to the richness of the discussion of the barriers encountered in clinical settings in these countries.

### 4.2. Implications and Further Research 

The perspectives of “ordinary” older adults and health professionals explored in this study suggest that one way to build HL is through interventions targeted at older adults’ everyday lives as well as interaction settings with health professionals and the broader health care systems that they have to navigate. We therefore encourage public health professionals to develop needs-based HL initiatives aimed at contextual elements to support older adults in using their HL abilities, for example, community-based interventions. Such initiatives should focus on exploring the age-specific needs and context of a target population, to identify their needs, and to foreground these needs in an intervention (see Paasche-Orlow et al. [42]). These initiatives could focus on, for instance, different perspectives as to how one could interact with health professionals or the importance of engaging in everyday routines to stay healthy. HL initiatives such as these are likely to have the most relevance for helping older adults gain control over their own health and well-being in a comprehensive manner and to build their CHL abilities. Such initiatives would add a different perspective to HL beyond that of researchers and practitioners. In addition, we encourage future research on HL that delves deeper into how older adults manage their health in their everyday lives. A research focus on this context will teach us more about how health literate “ordinary” people are and how they use their valuable skills to stay healthy.

## 5. Conclusions

This article aimed to explore and compare the context-specific lay perspectives on HL of older adults and health professionals in Greece, Hungary, and the Netherlands. An overarching finding of our article is that HL reflects the demands placed on older adults when they are managing their health. In their experience, these demands are placed upon them by healthcare professionals, the healthcare system, as well as their everyday lives. In describing these demands, our participants reported a strong need to be in control of their own health and well-being. Our main conclusion is that the most important aspect of HL for older adults in Greece, Hungary, and the Netherlands is to be empowered to maintain their own health and well-being within interactions with healthcare professionals, the broader healthcare system, as well as their everyday lives. Achieving this is often difficult, as the needs of older adults are specific and depend on, for instance, the sociocultural context in which they live, as well as their individual health and social circumstances.

## Figures and Tables

**Table 1:** Number of focus group discussions (FGDs) and participants in the three countries.

	Greece	Hungary	The Netherlands
Older adults (participants/FGD)	3 * (4–10)	2 (8)	2 (7)
Health professionals (participants/FGD)	2 (4–7)	2 (6–8)	1 (5)

* One pilot-focused group discussion (FGD) was conducted. As the data from that group corresponded with that of the other FGDs and therefore enriched the total dataset, we decided to include it in the analysis.

**Table 2:** Strategies for participant recruitment in the three countries.

Participants	Greece	Hungary	The Netherlands
Older adults	Local high school of Oinofita	Community websites, e.g., iwiw.hu and webbeteg.hu	Support and information center in Vinkhuizen,
	Two public day care centers for senior citizens in Aigaleo	Hospitals and out-patient departments	community centers in Paddepoel, Vinkhuizen and Korreweg, and Think-tank 60+ North-Netherlands
Health professionals	Primary Health Center of Social Security Organisation in Nea Smyrni	Rehabilitation centers, home nursing organizations, social welfare homes, and physician database of Szinapszis	Municipal Health Service in Groningen and elderly home care organizations

**Table 3:** Characteristics of the participating older adults.

Characteristics	Greece	Hungary	The Netherlands
N (Total)	20	16	14
Age Range (Median) (Years)	52–82 (69)	59–73 (62.5)	54–76 (62)
Gender (n)			
female	14	14	8
male	6	2	6
Retired			
yes	20	14	7
no	0	2	7
Health problems (n)			
none	2	0	7
single	10	2	1
multiple	8	14	6
Perceived health (n)			
excellent	0	0	0
good	6	6	8
fair	10	7	5
poor	4	3	1
Education			
none			2
primary school	1		
secondary school	15		6
primary vocational (lower education after high school)	4	16	5
secondary vocational (mid-high education after high school)			1

**Table 4:** Characteristics of the participating health professionals.

Characteristics	Greece	Hungary	The Netherlands
N (Total)	11	14	5
Types of nurses/social workers (n)			
Clinical nurse	7		
Community based nurse		4	1
Rehabilitation practitioner		3	
Community worker		1	1
Educator healthy lifestyle			2
Caretaker day care			1
Types of GPs/specialists (n)			
Physician/specialist	4		
General practitioner (GP)		4	
Rheumatologist		2	

**Table 5:** Summary of results.

Being in Control of Health and Well-Being		
Theme	Subtheme	Findings (General and Per Country)
Interactions with health professionals	The consultation room	All countries: important setting
		NL: being assertive; trustful relationship important
		HU: afraid to ask questions; professionals only respond; difficulties getting professionals’ attention
		GR: little share in conversation as norm; respect for doctors; difficulties opening up
	Consultation times	All countries: too short
Quality of health care system	Healthcare system	NL: Public health care but compulsory insurance to be paid with own contribution
		HU: Public, tax-funded health care system
		GR: Combination of public and private: pensioners are covered but pay 10% of costs
	Quality of care	NL: Quality is high, but care is expensive
		HU: Not being able to pay customary gifts is a reason to avoid healthcare
		GR: Quality of private care is high but too expensive
	Affordability	All countries: healthcare is too expensive
	Waiting lists	HU + GR: waiting lists for appointments are long
	Coordination of care and information	NL: fragmentation of services
		HU + GR: lack of coordination, resulting in inefficiency
		HU: health information is not aligned
Everyday lives	Caring for oneself	NL: Making choices oneself
		GR + HU: pressure on family
		GR + HU: fear of becoming a (financial) burden for family
		GR: Expectation that children will provide care
	Engaging in activities that support health and well-being	All countries: involvement in family life
		NL + HU: engagement in organized activities
		NL: engagement in (paid) work

## Supplementary Materials

The following are available online at https://www.mdpi.com/1660-4601/17/7/2411/s1.
