# Supplementary material for: Health Literacy in the Everyday Lives of Older Adults in Greece, Hungary, and the Netherlands
Source: Int J Environ Res Public Health. 2020 Apr 2;17(7):2411. doi: 10.3390/ijerph17072411 (PMC7177367; doi:10.3390/ijerph17072411)
Supplement: Supplementary file 1 [file ijerph-17-02411-s001.zip › ijerph-761650-SI/ijerph-761650 - Appendix A - Discussion Guide_Older Adults.pdf]

# Appendix A: Focus group discussion guide Older Adults

Date, time: …………………………………………………………………………………………

Location: ……………………………………………………………………………………………

Moderator: …………………………………………………………………………………………

Note taker: …………………………………………………………………………………………

Remarks: ……………………………………………………………………………………………

---

## Facilities

Refreshments and small snacks will be provided to participants before/during/after the FGD, travel costs will be reimbursed and participants will receive a small gift (for example: a voucher to spend at a local shop or food counter or a local souvenir) to acknowledge their participation at the end of the FGD.

## Role of researchers

Moderator: introduce the study, leads the discussion and motivates participants to speak.
Note taker: Responsibility **recording**, keeps a **field diary** that includes the process, interactions, atmosphere, and positionality of the FGD. Please try to capture who says what. Prepare **cards** for the ranking exercise in TOPIC 4. The note taker also supports in the poster/leaflet and ranking exercises in TOPIC 3 and 4, resp.).

## Informed consent

Make sure that you have introduced the study and explained the FGD procedures according to the introduction sheet and that the all participants have completed the consent form.

## The AIMS of the FGD are to:

- explore health and wellbeing in the context of older adults' lives to develop an understanding of people's motivation for their actions/non-actions (these are potentially related to health literacy/ interventions): TOPIC 1 AND 4
- explore ideas, needs and perspectives of older adults with low health literacy on accessing, understanding and use of health services: TOPIC 2
- explore ideas, needs and perspectives of older adults with low health literacy on accessing, understanding and use of health information and health literacy interventions that target older adults: TOPIC 3

- explore individual and environmental factors that may influence health literacy and related interventions, for example: high health literate partners/caretakers or resources to access health information or health services: TOPIC 1, 2 AND 3

## Introduction

The aim of the study and how the FGD will work you have explained to the participants when filling in the baseline questionnaire and the consent form. Repeat study purpose and FGD instructions briefly if the FGD does not take place on the same day that you filled in the forms (e.g. that the FGD will take about 1,5 hour). Also ask for additional individual verbal consent at the time of the FGD (on record) to ensure that participants have not changed their minds to participate.

## Introductory questions

Aims: a) To allow each participant to introduce themselves to facilitators and group; b) to give the moderator time to match the background information to each participant in the group.
Moderator: "Before we start I would like to ask you to introduce yourselves to the group". Make a round and ask all participants individually to introduce themselves by answering the following questions:

- What is your name?
- Why have you come along?
- Do you know any of the other people in the group? Who? How do you know them?

## TOPIC 1: Perception of health and wellbeing

Aims: 1) explore health and wellbeing in the context of older adults' lives to develop an understanding of people's motivation for their actions/non-actions (potentially related to health literacy/interventions); 2) explore individual and environmental factors that may influence health literacy and related interventions.
Moderator: "I would like to start the discussion with a very general question:"

**1. Can you please tell me what is important for you in life?**

*Probes:*

- *Health*
- *Social contacts,*
- *Wellbeing/quality of life*
- *Participation in society*
- *Other (for example: good financial situation)*

Moderator: Now you will discuss the different factors of importance that were mentioned in the group. Repeat questions 2, 3 and 4 for all factors that were discussed in question 1.

**2. You just told me: ["*health/social contacts/wellbeing/participation in society/other"*] is important for me"; can you explain what you mean by this?**

*Probes with health:*
- *physical health (what, why)*
- *mental health (what, why)*
- *own/others health (who)*
- *how would you describe your current health?*

*Probes with social contacts:*
- *with whom: children, grandchildren, other relatives, friends, neighbours, social workers, other*
- *why: prevention from isolation/loneliness/boredom*
- *describe such a meeting*

*Probes with wellbeing/quality of life:*
- *stress (what, when, why)*
- *loneliness (what, when, why)*
- *boredom (how, when, why)*
- *money*
- *access to health systems*
- *confidence/ self-management*
- *satisfaction*
- *happiness*
- *religion/ spirituality*

*Probes with participation in society:*
- *independence*
- *mobility*
- *availability and/or accessibility of facilities*
- *availability and/or accessibility of health care or other services*
- *respect/appreciation by other people*
- *prevention from social isolation/loneliness/boredom*

*Probe with 'other' factors that participants have mentioned as being important in their lives.*

**3. Can you tell me what you do to [*stay healthy/have social contacts/create greater wellbeing/participate in society/'other'*]?**

*Probes with health:*
- *eat a healthy diet*
- *physical activity (walking, cycling)*
- *sports*
- *social activities*
- *nothing: why not?*

*Probes with social contacts:*
- *home visits: with whom*

- *social activities: what kind, where, how often*
- *how: face to face, telephone, email, writing letters*

*Probes with wellbeing:*
- *hobbies (what)*
- *set goals (what)*
- *fill day with activities (what)*
- *professional aid (what, from whom)*
- *church visits*
- *spiritual activities*
- *sports/ dance*
- *social activities/ family*
- *reading newspapers/ crosswords/ …*

*Probes with participation in society:*
- *active or passive participation (what, how)*
- *work (paid/voluntary)*
- *membership of clubs/societies*
- *excursions/ holidays*
- *grocery shopping*
- *health care/cure related activities (e.g. rehabilitation/therapy)*

## TOPIC 2: Coping with health and experiencing health services

Aims: 1) explore ideas, needs and perspectives of older adults with low health literacy on accessing, understanding and use of health services; 2) explore individual and environmental factors that may influence health literacy and related interventions.
Note 1: This could be a time consuming section in the group of people with complex health issues.
Note 2: If people feel uncomfortable with speaking about their own health problems, the moderator can decide to switch to the experiences of people the participants know.
Moderator: "We have just discussed things that are important for you. Now I would like to talk about how you cope with your health and your experiences with health services."
"As people grow older they experience that certain activities become more difficult or that our body or mind does not always do all things we would like to anymore, for example: we get *difficulties with walking, we start to forget things, our eyes become less good, our hearing declines or we get a chronic disease, such as diabetes.* Also, our *environment* could change, such as the people we have around us. Now I would like to ask you…"

**4. What kind of health issues do you think senior citizens generally face?**
*Probes:*
- *physical issues: eyes, hearing, walking, disease (type?)*
- *mental issues: forgetfulness, dementia, depression, stress*
- *social issues: loneliness, boredom, discrimination, stress, family problems*

- *benefits to growing older: more time, space, less responsibilities (type?)*

**5. Can you tell me about the health issues YOU experience yourselves?**

*Probes:*
- *physical issues: eyes, hearing, walking, disease (type?)*
- *mental issues: forgetfulness, dementia, depression, stress*
- *social issues: loneliness, boredom, discrimination, stress, family problems*

**6. Please tell me about the impact these health issues have on your daily life?**

*Probes:*
- *Immobility*
- *solitary life*
- *lonelinessfeeling discriminated*
- *dependence on other people*
- *experience of change (physical deterioration, housing)*
- *expectations of future health*
- *consideration of being healthy, difficulty taking medicine, following medical advice, knowing the appropriate place to go to receive medical care*
- *financial problems*

**7. How do you attempt to cope with these issues?**

*Probes:*
- *accepting (easy/difficult)*
- *make your own choice/others choose for you what to do*
- *seeking for help from professional or family/friends/neighbour (=accessing health service: how?)*
- *seek for information about the issue (=accessing health information: what type, how?)*
- *both seek for information AND help from a professional or family or friends or neighbours*
- *prevention strategies (healthy lifestyle, being part of a lobbying group or residents association)*
- *do nothing: why? (=NOT accessing health information nor care/cure)*
- *other*

**8. What health services do you use?**

*Probes:*
- *General practitioner*
- *Specialist*
- *Psychologist or other mental help*
- *Physio/rehabilitation therapy*
- *Elderly home care (cleaning, getting dressed/showered, support in medicine use, etc)*
- *Social aid (administration etc)*
- *Complementary/Alternative Medicine (CAMS in UK) e.g. herbal remedies, acupuncture, reflexology, homeopathy etc)*

**9. How do you experience these services?**

*Probes:*
- *overall satisfaction: good or bad*
- *experience of patient-professional relationship: friendly, careful, helpful, qualified*
- *communication with health professional: clear, sufficient, they listen to me, time/openness for questions, they are patient with me (=understanding communication with health professionals)*

## TOPIC 3: Health information and interventions

Aims: 1) explore ideas, needs and perspectives of older adults with low health literacy on accessing, understanding and use of health information and health literacy interventions that target older adults; 2) explore individual and environmental factors that may influence health literacy and related interventions.

Moderator: "Now we talked about how you cope with health and how you experience health services. I would now like to discuss how you receive information about getting and staying healthy and your understanding of this information".

First show the poster/leaflet that contains the health care message, and ask Q10-13. Subsequently show the poster/leaflet with the message about prevention. Ask Q10-13 again.

**10. Now I would like to show you this poster/leaflet. How familiar is this poster/leaflet, or similar posters/leaflets, to you?**

*Probes:*
- *Never seen it*
- *Recognize this poster/leaflet (from wher:tv, doctor, hospital, etc?)*
- *Seen familiar posters/leaflets (about what?)*
- *Where have you seen it (places: health service, community centre, supermarket, internet, newspaper)*

**11. Could you explain to me what the poster/leaflet shows?**

*Probes:*
- *topic*
- *message*
- *aim of the poster*
- *population group targeted (for you or not?)*

**12. And if you see a poster/leaflet like this one, how do you respond to it?**

*Probes:*
- *do not respond: why not? (particular message not for me, type of information not for me, not able to respond, not my own choice: why?)*
- *feel the need to take action, but I do not take action: why? (not able to respond, not my own choice: why?)*
- *take action: why/how/what?*
- *Does not apply to me*
- *Makes me feel helpless / depressed / guilty etc*

**13. What do you find attractive to this poster/leaflet?**

*Probes:*

- *not attractive: why not? (difficult language to understand, text too small, colours not clear, …)*
- *pictures: what/why?*
- *text: what/why?*
- *combination pictures/text: what/why?*
- *colours: what/why?*

**14. How do you prefer to receive the information you need about getting better if you are ill and staying healthy: in the form of a poster/leaflet like these or in other ways, for example through your GP?**

*Probes:*

- *Posters/leaflets like these*
- *Posters/leaflets, but differently presented (how?)*
- *Other: formal contacts (health services, such as GP: why?)*
- *Other: informal contacts (family, friends, neighbours, caretakers: why?)*
- *Other: media (radio, tv, internet, mobile phone)*

**15. What do you do if you feel the need for particular information about your health?**

*Probes:*

- *Never feel the need for health information (why not?) (all necessary info available, understandable and accessible/do not use health info?)*
- *Do nothing (why not?)*
- *Search for information (how/where/for what type of info?)*
- *Ask someone (who?) (family, friend, neighbor, caretaker, health professional)*

## TOPIC 4: Ranking issues about health and wellbeing

Aim: a) explore health and wellbeing in the context of older adults' lives to develop an understanding of people's motivation for their actions/non-actions; b) to wrap up the discussion.

<u>Moderator: "We have spoken about a lot of topics related to your health, health services and information about health. We have come towards the end of our discussion now. "</u>

"The last thing I would like to ask you is the following. My colleague [note taker] has developed **pictures** of different topics that we have just talked about in our discussion on these cards. " [show cards of different topics that were mentioned as important for health, wellbeing, health literacy and interventions. We have several cards with issues that we expect to hear in the discussion prepared already. In addition, the note taker draws pictures of certain issues raised in the discussion that have not been presented on a card yet. He/she tries to use a similar drawing style. Topics on the cards are prominent issues that have been mentioned by the participants in the discussion. The cards contain **pictures** in a **simple drawing style** and can range from broad to more specific topics, for example: (examples of) physical, mental and/or social health, such as being able to walk, being clear in the mind or contacts with family, healthy diet, easy access to health care, particular health message, communication with health staff, etc) "Now I will ask you one by one…"

**16. Could you tell me which card is most important for you?**

*Probes:*

- *Describe why*
- *Describe why the others not*
- *Easy or difficult to choose*

## Closing the discussion

Moderator: "We have discussed a lot of topics, ranging from your own health to your experiences with health services and information about health care and prevention of diseases. I would like to ask you whether there are any more important things related to these topics that you feel we did not discuss, and that you would like to share in the discussion?"
Moderator: "I would like to end the discussion now. How have you experienced the discussion?"
**Moderator/note-taker: hand the gifts and thanks them for their participation**

**Final announcement**
Moderator: "In the next weeks I will write out this discussion and conduct more group discussions with other people. Besides, I will analyze the findings of the group discussions. In April I would like to come back to you, and have a follow-up discussion with you, to share the results from the discussions, because we find it very important that our participants can recognize themselves in the final results. In this follow-up discussion I will tell you about the things we learned from the different group discussions. And, more important, I would then like to ask you whether you recognize your own stories in these findings, and whether we have missed important things. So, you contribution will be very valuable, because it will probably change our initial results".

Ask all participants individually:
- if they agree on participating in this follow-up discussion in April, and if they could be contacted by phone by April to set a date, time and location.
- If they would like to receive further results of the IROHLA study and how (email/post).

Moderator: "I would like to thank you all very much for your participation in this study and I am looking forward seeing you again in April for the next discussion".
