# Supplementary material for: Health Literacy in the Everyday Lives of Older Adults in Greece, Hungary, and the Netherlands
Source: Int J Environ Res Public Health. 2020 Apr 2;17(7):2411. doi: 10.3390/ijerph17072411 (PMC7177367; doi:10.3390/ijerph17072411)
Supplement: Supplementary file 1 [file ijerph-17-02411-s001.zip › ijerph-761650-SI/ijerph-761650 - Appendix B - Discussion Guide_Health professionals.pdf]

Appendix B: Focus group discussion guide Health Professionals

Date, time: ……………………………………………………………………………………………

Location: ……………………………………………………………………………………………

Moderator: …………………………………………………………………………………………

Note taker: …………………………………………………………………………………………

Remarks: ……………………………………………………………………………………………

Facilities

Refreshments and small snacks will be provided to participants before/during/after the FGD, travel costs will be reimbursed and participants will receive a small gift (for example: a local souvenir) to acknowledge their participation at the end of the FGD.

Role of researchers

Moderator: introduce the study, leads the discussion and motivates participants to speak.
Note taker: Responsibility **recording**, keeps a **field diary** that includes the process, interactions, atmosphere, and positionality of the FGD. Please try to capture who says what. The note taker also supports in the poster 'exercise'.

Informed consent

Make sure that you have introduced the study and explained the FGD procedures according to the introduction sheet and that the all participants have completed the consent form.

The AIMS of the FGD are to:

- explore the perceptions of health professionals on what health literacy means TOPIC 1
- explore the perceptions of health professionals on how older adults with low health literacy navigate in the health care system TOPIC 2
- explore the perceptions of health professionals on how older adults with low health literacy use information on their health and wellbeing TOPIC 3
- explore how health professionals perceive the interventions aimed at older adults with low health literacy TOPIC 4

Introduction

The aim of the study and how the FGD will work you have explained to the participants when filling in the consent form. Repeat study purpose and FGD instructions briefly if the FGD does

not take place on the same day that you filled in the forms (e.g. that the FGD will take about 1,5 hour). Also ask for additional individual verbal consent at the time of the FGD (on record) to ensure that participants have not changed their minds to participate.

Introductory questions

Aims: a) To allow each participant to introduce themselves to facilitators and group; b) to give the moderator time to match the background information, which participants filled in on the survey form, to each participant in the group.

Moderator: "Before we start I would like to ask you to introduce yourselves to the group". Make a round and ask all participants individually to introduce themselves by answering the following questions:

- What is your name? And your age?
- What is your occupation?
- What education have you pursued?
- Why have you come along?
- Do you know any of the other people in the group? Who? How do you know them?

TOPIC 1: HP's understanding of health literacy

Aim: to explore the participants' ideas on what they mean with the term health literacy
Moderator: "In the introduction, you all told us a little bit about your profession. You all work with older adults who have a low health literacy (amongst others). First, I am interested in your understanding of health literacy." Therefore my first question to you is…"

1. **How would you describe the term 'health literacy'? (Or: when you hear the term 'health literacy', what do you think it means?)**

Probes:
- accessing health information on care/cure/prevention and the health care system
- understanding health information on care/cure/prevention and the health care system
- processing health information on care/cure/prevention and the health care system
- using/applying health information on care/cure/prevention and the health care system
- comprehensibility of communication between health professional and patient
- knowledge/competences/motivation to take action related to health information/ health care
- examples of health literacy (related topics/issues), e.g. adherence to prescribed medication
- different levels of health literacy: functional, interactive, critical

TOPIC 2: Older adults with low HL in the health care/social system

Aim: to explore the perceptions of health professionals on how older adults with low health literacy navigate in the health care system

Moderator: "We have just talked about what you mean by health literacy. (Give a description of health literacy if participants did not know the term)In our study we are interested in older adults who have a low health literacy. Now I would like to talk about how you think these older adults navigate in the health care and/or social system."

2. **What kind of health issues do you think older adults with low HL typically face?**
   Probes:
- *physical issues: eyes, hearing, walking, disease (type?)*
- *mental issues: forgetfulness, dementia, depression, stress*
- *social issues: loneliness, boredom, discrimination, stress, family problems*
- *benefits to growing older: more time, space, less responsibilities (type?)*

3. **What impact do you think these health issues have on the daily lives of older adults with low HL?**
   Probes:
- *Immobility*
- *solitary life*
- *loneliness/feeling discriminated*
- *stress/depression*
- *boredom*
- *dependence on other people*
- *experience of change (physical deterioration, housing)*
- *expectations of future health*
- *consideration of being healthy, difficulty taking medicine, following medical advice, knowing the appropriate place to go to receive medical care*
- *financial problems*

4. **Can you tell me when you think these senior citizens come to see you as a health/ social work professional?**
   Probes:
- *Type of complaints*
- *Whose initiative? Family, other health professional*
- *Too early/ too late?*
- *Further referral to other health/social work professionals?*

TOPIC 3: Older adults and their use of health information

Aim: to explore the perceptions of health professionals on how older adults with low health literacy use information on their health and wellbeing

5. **How do you experience the communication with older adults who have a low health literacy?**
   Probes:
- *Differences in communication with younger and older adults in general?*

- *Experience a difference between older adults with low HL and older adults with higher HL (easier/more difficult)? How acting on it?*
- *I do realize that there are older adults with lower HL than others, but I do not act on it (why)*
- *When talking to clients, I am not aware that some older people have lower health literacy than others*
- *I experience no difference in health literacy among older adults*
- *They ask for info?*
- *I give info to them without them having asked for it*

6. **What kind of information about prevention/care/cure do you typically give to older adults with a low health literacy in your professional practice?**
   Probes:
- *Spoken information*
- *Written information, such as leaflets*
- *Visual information, such as leaflets*
- *Referral to websites*
- *Referral to patient organisations*
- *Differences between older adults with higher and lower HL?*

7. **What do you think they do with your information?**
   Probes:
- *do hear it?*
- *do understand it?*
- *process it/interpret it well*
- *will apply it into their lives?*
- *Are not interested in info like this?*

Now, I would like to discuss two posters with you. They contain visual and written information about health. The first one is on a prevention topic, the second one on a care-related topic. First show the poster that contains the health care message, and ask Q8-10. Subsequently show the poster/leaflet with the message about prevention. Ask Q8-10 again.

8. **Now I would like to show you this poster. What message do you think the poster wants to convey?**
   Probes:
- *topic*
- *message*
- *aim of the poster*
- *population group targeted*

9. **Imagine you show the poster to one of your clients, an older adult with a low level of health literacy. How do you think such a person would react to it?**
   Probes:

- *do not respond: why not? (particular message not for me, type of information not for them, not able to respond, not their own choice)*
- *feel the need to take action, but do not take action: why? (not able to respond, not their own choice: why?)*
- *take action: why/how/what?*
- *Does not apply to them*
- *Makes them feel helpless / depressed / guilty etc*

10. What do you think is a good way to give older adults with low health literacy information about their health?

Probes:
- *Posters/leaflets like these*
- *Posters/leaflets, but differently presented (how?)*
- *Other: formal contacts (health services, such as GP: why?)*
- *Other: informal contacts (family, friends, neighbours, caretakers: why?)*
- *Other: media (radio, tv, internet, mobile phone)*

TOPIC 4: Health interventions targeting older adults with low health literacy

Aim: to explore how health professionals perceive interventions aimed at older adults with low health literacy

11. Are you aware of any health interventions, both in prevention, care and cure, which are aimed specifically at older adults who have a low health literacy?

Probes:
- *Yes: ask which interventions*
- *No: ask whether they would think such interventions to be useful. What could be the result of such interventions?*
- *Further probing with examples of interventions: television campaigns, e-health, local community projects.*
    - *Where?*
    - *Targeted at whom?*
    - *Initiators?*
    - *Measures taken?*
    - *Effects? Does HL improve, especially with older adults who have a low HL?*
    - *Success factors?*
    - *Factors that explain failure?*

Closing the discussion

12. What do you believe that you as a health/social care professional could do specifically for older adults with low health literacy?

Probes:
- *Using simple wording when talking to people*
- *Explaining things more than once*

- *Showing special written information (large font, simple sentences)*
- *Refer them to other professionals? Which ones*

Moderator: "We have discussed a lot of topics, ranging from health literacy in general to your own communication with older adults who have a low HL. I would like to ask you whether there are any more important things related to these topics that you feel we did not discuss, and that you would like to share in the discussion?"

Moderator: "I would like to end the discussion now. How have you experienced the discussion?"

Moderator/note-taker: hand the gifts and thanks them for their participation

Final announcement

Moderator: "In the next weeks I will write out this discussion and conduct more group discussions with other professionals as well as older adults. Besides, I will analyze the findings of the group discussions. In April I would like to come back to you, and have a follow-up discussion with you, to share the results from the discussions. Reason for this is that we find it very important that our participants can recognize themselves in the final results. In this follow-up discussion I will tell you about the things we learned from the different group discussions. And, more important, I would then like to ask you whether you recognize your own stories in these findings, and whether we have missed important things. So, you contribution will be very valuable, because it will probably change our initial results".

Ask all participants individually:

- if they agree on participating in this follow-up discussion in April, and if they could be contacted by phone by April to set a date, time and location.
- If they would like to receive further results of the IROHLA study and how (email/post).

Moderator: "I would like to thank you all very much for your participation in this study and I am looking forward seeing you again in April for the next discussion".
